# A Simple Co-culture System for Generation of Embryonic Stem-Like Cells From Testis

**DOI:** 10.5812/ircmj.4051

**Published:** 2012-12-06

**Authors:** Maryam Nazm Bojnordi, Mansoureh Movahedin, Taki Tiraihi, Mohamad Javan

**Affiliations:** 1 Department of Anatomical Sciences, Medical Sciences Faculty, Tarbiat Modares University, Tehran, IR Iran; 2 Department of Physiology, Medical Sciences Faculty, Tarbiat Modares University, Tehran, IR Iran

**Keywords:** Spermatogonia, Reprogramming, Testis

## Abstract

**Background:**

New research proposes the pluripotency of spermatogonial cells obtained from testis. These spermatogonia-derived stem cells are called embryonic stem-like cells that express embryonic stem cell markers and differentiate to the three germ layers.

**Objectives:**

The aim of the present study was to generate embryonic stem-like cells from neonatal mouse testis.

**Materials and Methods:**

The Testis cells were collected from neonatal mouse. After decapsulation, testis was mechanically dissected and dissociated via a two-step mechanical and enzymatic digestion. The spermatogonia and sertoli cells were cultured together in Dulbecco’s modified Eagle’s medium (DMEM) supplemented with 15% FBS and LIF. Before one week, several small spermatogonia colonies were observed on top of the monolayer of sertoli cells. These colonies were passaged every four days. ES-Like cells colonies that resembled ES cell was appeared within 2-3 weeks (at passages 5). Real time PCR was performed to analyze the expression of a subset of pluripotency markers, as well as germ cell-specific genes. ES Like cells were confirmed with SSEA1, SOX2 and Oct4 immunofluorescence stainng as pluripotency markers.

**Results:**

The Results showed that at fifth passages, the pluripotency genes; Nanog and c-myc have significant increase in ES-Like cells in compare with spermatogonia cells, whereas the spermatogonial markers; Stra8, mvh, and piwill2 became downregulated. In addition to these pluripotency genes, the ES cell marker SSEA-1, SOX2 and Oct4 were expressed in the ES-like cells, similar to ES cells.

**Conclusions:**

This researh indicates pluripotency evidence of ES-like cells derived from testis. ES-like cells shows some molecular characteristics with embryonic stem cells.

## 1. Background

Pluripotent stem cells are characterized by their extensive proliferation and self-renewal ability and they can differentiate into many different cell types, this property makes them a renewable source which are usable for the treatment of disease and developmental research (1, 2).Different type of pluripotent stem cell is recognized in mammals. Embryonic stem (ES) cells originated are generated from blastocyst inner cell mass. Embryonic germ (EG) cells are derived by culturing primordial germ cells. Embryonal carcinoma (EC) cells are derived from testicular tumors (3, 4).The recent research shows that embryonic stem-like cells can also be generated from mouse spermatogonial stem cells (SSCs) under culture condition. The origin of spermatogonial stem cells is from primordial germ cells (PGCs), that migrate to the early gonadal ridge during organogenesis (5).

SSCs have the ability of spermatogenesis which produces gamet, in addition these cells can transit to pluripotent cells which differentiate into all three germ layers (6). They will be usable for basic research and studying the differentiation mechanisms of stem cell. They offer a new stem cells source for treatment of disease in human regenerative medicine (7). Some criteria, such as differentiation into three germ layers and contribution to chimeras, demonstrated the pluripotency of mouse spermatogonia-derived ES like cells (8, 9).Among different sources for cell-based therapy such as cell lines, bone marrow, hematopoietic or embryonic stem cells, some evidence have suggested extensive proliferation activity and pluripotency of spermatogonial stem cells (10-12).

These cells can form embryonic stem-like cells and embryoid body structure in vitro that can differentiate to three germ layers (13, 14). This novel pluripotent stem cells doesn’t have the ethical limitation associated with ES cell and decrease immune rejection problems of transplantation therapies so it can be useful in regenerative medicine (13). The developmental models for studying genetical diseases is possible by generation of pluripotent stem cells from patients with genetic disorders (14).These characteristics proposes the therapeutic use of spermatogonial stem cells as a new and appropriate source for future stem cell-based therapies and regenerative medicine (13).

## 2. Objectives

According to pervious reports showed pluripotency of testis, we designed a simple co-culture of spermatogonia with sertoli cells for derivation of ES-Like cells from neonatal mouse spermatogonia cells in vitro.

## 3. Materials and Methods

Testis cells were collected from neonatal mouse (2–4 days old). After decapsulation of the testis, tissue was mechanically dissected and dissociated via a two-step mechanical and enzymatic digestion with DMEM medium which contained; 0.5 mg/ml collagenase-dispase, 0.5 mg/ml trypsin and 0.05 mg/ml DNase, (with shaking and pipetting) at 37°C for 30-45 min.

### 3.1. Co-culture of SpermatogonialCells With Sertoli Cells

The obtained cells that included spermatogonia and sertoli cells were incubated together at 37°C and 5% CO2, in a humidified atmosphere, they were cultured in Dulbecco’s modified Eagle’s medium (DMEM) (Invitrogen) supplemented with 15% FBS, 1 mMLglutamine (Invitrogen), 0.1 mM nonessential amino acids (Invitrogen), 0.1 mM-mercaptoethanol and LIF. Two days postplating, most testicular cells were attached to the growing surface, and medium was changed. Before one week, several small spermatogonia colonies were observed on top of the monolayer of sertoli cells. These colonies were passaged every four days. Finally, ES-Like cells colonies that resembled ES cell was appeared within 2-3 weeks (at passages 5). The number of the cells was determined using a hematocytometer before culture. Cell viability was evaluated by means of dye exclusion test (trypan blue solution). ES-Like cells were confirmed with Real Time- PCR and Immunoflurcent staining. Embryonic stem cells considered as a control group.

### 3.2. Embryoid Bodies

EBs was created using hanging drop method. ES-Like cells were lifted, washed, and resuspended in LIF-free DMEM supplemented with 5% FBS to a concentration of 2000 cells per 20μl. Twenty micro liter drops of the suspension were placed on the lid of a 10-cm plastic culture dish (Falcon). The lid was turned upside down and placed on the bottom part of dish, which was filled with sterile water, creating hanging drops. The dishes were incubated at 37°C in 5% CO2. EBs were cultured for 48 h.

### 3.3. Immunofluorescence Staining of ES-Like Cells Colonies

For immunofluorescence, the cells were fixed in 4% paraformaldehyde (PFA) in PBS (pH 7.4) for 20 minutes. The cells were washed twice with PBS/0.1% Tween-20 to remove residual fixative and incubated in 1%Triton X in PBS for 30 minutes, prior to blocking in 4% normal goatserum in PBS for 30 minutes followed by incubation with antibody solution overnight at 4°C. Primary antibodies included: stage-specific embryonic antigen 1 (SSEA1) (1:200; Abcam), SOX2 (1:500; Abcam) and Oct4 (1:200; Abcam). In the following day, the cells were washed twice with PBS, and incubated with appropriate secondary antibody (1:1000; Abcam) in PBS. After two washes with PBS for 5 minutes, the cells were mounted with anti-fade mounting 4, 6-diamidino-2-phenylindole (DAPI)/PBS and viewed on a Leica microscope.

### 3.4. Gene Expression Analysis and Real Time PCR in ES-like Cells

Real time PCR was performed to analyze the expression of a subset of pluripotency markers, as well as germ cell-specific genes. Nanog and c-myc were evaluated as of pluripotency genes in ES-Like cells and the spermatogonial markers Stra8, mvh and Piwill2 were evaluated.

## 4. Results

**Figure 1:**
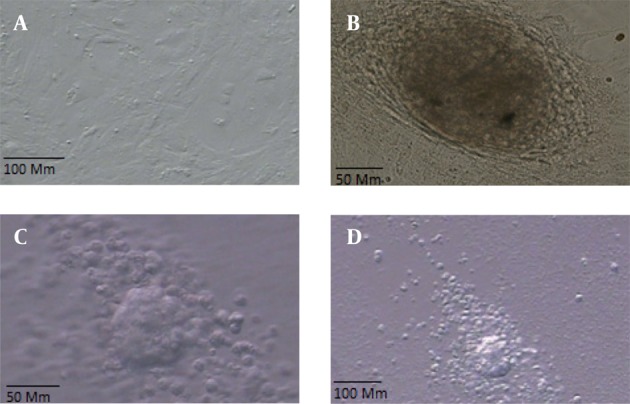
a: Sertoli Cells, b: The Morphology of a Spermatogonial Derived Colony Formed From Co-culturing of Spermatogonial Cells on a Monolayer of Sertoli Cells, c and d EB Formation Induced by Hanging Drop Method of ES-Like Cells After 2 Days in Vitro Culture

On the basis of our experience with isolating SCs from neonatal mice, we were able to identify the cells morphologically. Two days postplating, most testicular cells were attached to the growing surface. Approximately before 1 week, several small colonies were observed on top of the monolayer of testicular cells (Figure1,a-b). These colonies were passed every four days and finally ES-Like cells were appeared within 2-3 weeks (at fifth passages), colonies formed that resembled ES cell colonies.

### 4.1. Characterization of ES-Like Cells Using Immunostaining

**Figure 2:**
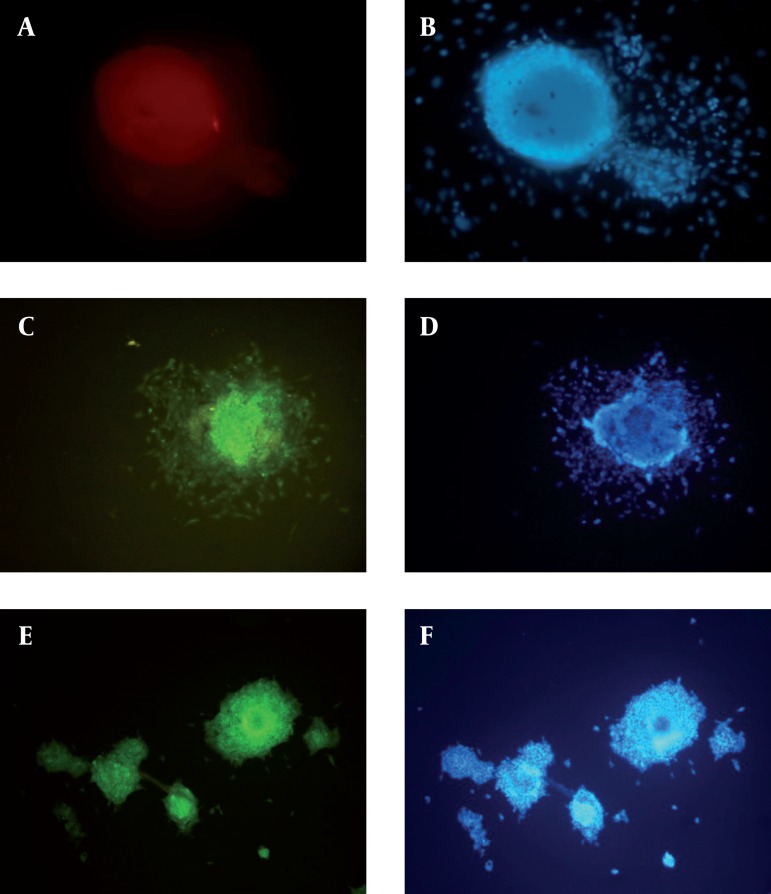
Immunostaining of SSEA-1, Oct4 and SOX2.(a): ES-Like Cells show Positive Reaction to SSEA-1, (b): DAPI for ES-Like Cells, (c): ES-Like Cells show Positive Reaction to Oct 4, (d): DAPI for ES-Like Cells, (e): ES-Like Cells show Positive Reaction to SOX2, (f): DAPI for ES-Like Cells Cells, Scale Bars: 50 μm.

For a more detailed examination of ES-like colonies, we performed immunocytochemical analysis and compared them with spermatogonial cells. ES-like cells expressed the cell-surface marker SSEA-1 (stage-specific embryonic antigen-1), SOX2 and Oct 4 that characterized undifferentiated mouse ESCs (Figure 2).

### 4.2. Real Time PCR

**Figure 3:**
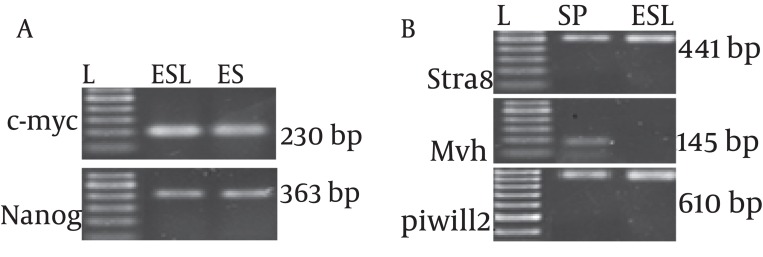
Polymerase Chain Reaction (PCR) for Identification of Pluripotency and Spermatogonial Markers; (a) C-myc and Nanog. (b) Stra8, Mvh and Piwill2. L: ladder,100 bp. ES: Embryonic Stem Cells, ESL: Embryonic Like Stem Cells, SC: Spermatogonia Cells

**Figure 4:**
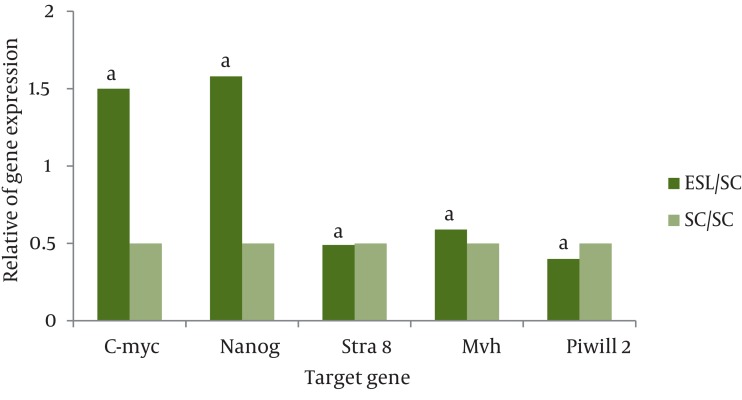
The Ratio of Pluripotency Genes Expression: Nanog, C-myc and Spermatogonial Genes: Stra 8, Mvh and Piwill2 in ES-Like cells in Compare with Spermatogonia Cells. a: Significant Increase or Decrease

**Figure 5:**
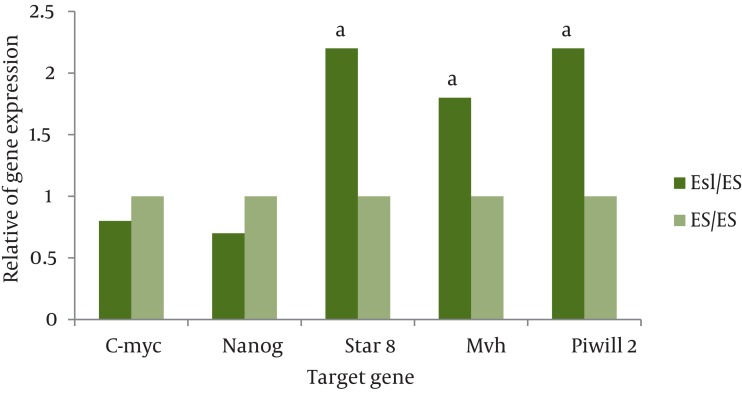
The Ratio of Pluripotency Genes Experssion: Nanog,C-myc and Spermatogonial Genes: Stra8, Mvh and piwill2 in ES-Like Cells in Compare With ES Cells. a: Significant Increase

Real time PCR was performed to analyze the expression of a subset of pluripotency markers, as well as germ-cell specific genes, in the isolated ES-Like cells. The results demonstrated that at fifth passages, in compare with spermatogonia cells, the pluripotency genes Nanog and C-myc has significant increase in ES-Like cells. Also the expression of Nanog and c-myc of these cells are similar to ES cells. Gene expression levels before and after the transition of cultured SCs to ES-like cells shows that the expression of spermatogonial markers Stra8, mvh, and piwill2 were down regulated, but they couldn’t reach to ES level genes expression (Figures 3, 4 and 5).

### 4.3. Formation of EB

The ability of EB formation in vitro is one of the characteristics of pluripotent ES-like cells. EB formation was detected after 2–4 days of hanging drop method (Figure 1, c-d).

## 5. Discussion

Generation of ES-like cells from testis by standard methods has many application in formation of cell lines from specific patient or disease that can be used in basic research and allowing sufficient cells for regenerative therapy (15). In addition, recent reports have documented the derivation of pluripotent stem cells that can differentiate to all cell types from both neonatal and adult mouse testis (16, 17). These studies show that pluripotent stem cells were generated under culture condition and without genetic modification. In this study, we isolated pluripotent cells from mouse testis that were cultured under ESC culture condition except sertoli cells was used as a feeder. The colonies with sharp border were similar to embryonic stem cell morphology and formed embryoid bodies structures, in suspension and these cells are ES-like cells.

The perspective of the results obtained in mice has clinical application in other species particularly in humans. There are different methods to induce ES-like cells; Kanatsu-Shinohara and Seandel used the same culture protocol, with some differences in feeder layers and time duration of emergence of ES cell-like cells (18, 19). Guan used culture protocol by adding GDNF in the first week of culture followed a simple medium with serum (8).

Time duration of emergence of ES-like colonies depends on the used culture system. Seandel used culture system with testicular peritubular and sertoli cells as a feeder layer. Guan method is the longest time in which no feeder layer was provided (8, 19).In contrast, Kanatsu-Shinohara found that if neonatal mouse SSCs were cultured in ES medium right from the start, ES-like colonies failed to form (20).The transition from cultured SSCs to ES-like cells is accompanied by extensive changes in gene expression. Our research proved that ES-like cells originated from the SCs show high levels of expression of the pluripotency genes Nanog and C-myc, similar to ES. Furthermore, germ cell-specific genes such as Stra8, Mvh and Piwill2 are down regulated which confirmed pervious results (20, 21).

These ES-like cells have the same molecular profiling with embryonic stem cells (22, 23). Our results indicate that SSCs can proliferate colonially on the feeder layer of sertoli cells and retain stable morphology. Co-culture spermatogonia cells with sertoli cell as a feeder layer provide a cell model for in vitro culture of spermatogonia cells. In fact, the importance of our simple method will be clear when we compare it with other complex and time consuming methods. ES-Like cells derived spermatogonial stem cells share many molecular and cellular characteristics with embryonic stem cells, on cellular level, these cells has the same morphology with a sharp edge and formation of embryoid body structure after culture in vitro. In contrast to ESCs, the use of SSCs for cell transplantation prepares sufficient cells and individual cell-based therapy, in addition, the ethical problem is avoided. These characteristics propose the therapeutic use of spermatogonial stem cells for regenerative medicine as a new and unlimited source.

## References

[1] Evans M, Hunter S (2002). Source and nature of embryonic stem cells.. C R Biol..

[2] Rossant J (2001). Stem cells from the Mammalian blastocyst.. Stem Cells..

[3] Turnpenny L, Cameron IT, Spalluto CM, Hanley KP, Wilson DI, Hanley NA (2005). Human embryonic germ cells for future neuronal replacement therapy.. Brain Res Bull..

[4] Baba S, Heike T, Umeda K, Iwasa T, Kaichi S, Hiraumi Y (2007). Generation of cardiac and endothelial cells from neonatal mouse testis-derived multipotent germline stem cells.. Stem Cells..

[5] Boulanger CA, Mack DL, Booth BW, Smith GH (2007). Interaction with the mammary microenvironment redirects spermatogenic cell fate in vivo.. Proc Natl Acad Sci U S A..

[6] de Rooij DG, Mizrak SC (2008). Deriving multipotent stem cells from mouse spermatogonial stem cells: a new tool for developmental and clinical research.. Development..

[7] Cyranoski D (2006). Stem cells from testes: could it work?. Nature..

[8] Guan K, Nayernia K, Maier LS, Wagner S, Dressel R, Lee JH (2006). Pluripotency of spermatogonial stem cells from adult mouse testis.. Nature..

[9] Kanatsu-Shinohara M, Lee J, Inoue K, Ogonuki N, Miki H, Toyokuni S (2008). Pluripotency of a single spermatogonial stem cell in mice.. Biol Reprod..

[10] Kanatsu-Shinohara M, Shinohara T (2006). The germ of pluripotency.. Nat Biotechnol..

[11] Takahashi K, Yamanaka S (2006). Induction of pluripotent stem cells from mouse embryonic and adult fibroblast cultures by defined factors.. Cell..

[12] Nayernia K (2007). Stem cells derived from testis show promise for treating a wide variety of medical conditions.. Cell Res..

[13] Nayernia K, Li M, Jaroszynski L, Khusainov R, Wulf G, Schwandt I (2004). Stem cell based therapeutical approach of male infertility by teratocarcinoma derived germ cells.. Hum Mol Genet..

[14] Park IH, Arora N, Huo H, Maherali N, Ahfeldt T, Shimamura A (2008). Disease-specific induced pluripotent stem cells.. Cell..

[15] Takehashi M, Kanatsu-Shinohara M, Miki H, Lee J, Kazuki Y, Inoue K (2007). Production of knockout mice by gene targeting in multipotent germline stem cells.. Dev Biol..

[16] Guan K, Wagner S, Unsold B, Maier LS, Kaiser D, Hemmerlein B (2007). Generation of functional cardiomyocytes from adult mouse spermatogonial stem cells.. Circ Res..

[17] Hofmann MC, Braydich-Stolle L, Dym M (2005). Isolation of male germ-line stem cells; influence of GDNF.. Dev Biol..

[18] Kanatsu-Shinohara M, Inoue K, Lee J, Yoshimoto M, Ogonuki N, Miki H (2004). Generation of pluripotent stem cells from neonatal mouse testis.. Cell..

[19] Seandel M, James D, Shmelkov SV, Falciatori I, Kim J, Chavala S (2007). Generation of functional multipotent adult stem cells from GPR125+ germline progenitors.. Nature..

[20] Kanatsu-Shinohara M, Ogonuki N, Inoue K, Miki H, Ogura A, Toyokuni S (2003). Long-term proliferation in culture and germline transmission of mouse male germline stem cells.. Biol Reprod..

[21] Kubota H, Brinster RL (2006). Technology insight: In vitro culture of spermatogonial stem cells and their potential therapeutic uses.. Nat Clin Pract Endocrinol Metab..

[22] Mardanpour P, Guan K, Nolte J, Lee JH, Hasenfuss G, Engel W (2008). Potency of germ cells and its relevance for regenerative medicine.. J Anat..

[23] Kossack N, Meneses J, Shefi S, Nguyen HN, Chavez S, Nicholas C (2009). Isolation and characterization of pluripotent human spermatogonial stem cell-derived cells.. Stem Cells..

